# The Lumber Supply of Washington Territory

**Published:** 1881-02

**Authors:** 


### THE LUMBER SUPPLY OF WASHINGTON TERRITORY.

Our Territory contains an area of almost 42,000,000 acres, including its mountain ranges, water courses, timber and prairie lands. Of these, probably 5,000,000 acres of the most available lands will average 50,000 feet of good timber to the acre, or in the aggregate will yield the enormous quantity of 250,000,000,000 feet. On the remaining 37,000,000 acres, probably as much more can be obtained with fair profit, a total aggregate of 500,000,000,000 feet of lumber. This is an enormous quantity, as much as has been consumed in all the shipyards, on all the house-building of the United States during the past 100 years. If properly conserved, the forests of Washington Territory will undoubtedly yield double the quantity named in the foregoing. To do this the ruthless destruction of timber that is constantly encountered must be stopped ; forest fires must end, and the saplings must be allowed to mature.—*Seattle Intelligencer*, *of Nov.* 20.

